# Crystal structures of 2,6-di­bromo-4-methyl­benzo­nitrile and 2,6-di­bromo-4-methyl­phenyl isocyanide

**DOI:** 10.1107/S2056989017016395

**Published:** 2017-11-21

**Authors:** Wayland E. Noland, Jessica E. Shudy, Janel L. Rieger, Zoe H. Tu, Kenneth J. Tritch

**Affiliations:** a Department of Chemistry, University of Minnesota, 207 Pleasant St SE, Minneapolis, MN 55455, USA

**Keywords:** crystal structure, nitrile, isocyanide, Br⋯Br contacts

## Abstract

In the title crystals, C_8_H_5_Br_2_N, which are isomorphous, the steric bulk of the methyl group causes neighboring mol­ecules to become mutually inclined. This prevents the formation of planar or nearly planar sheets, which were observed in the tri­chloro and tri­bromo analogs. Instead of CN/NC⋯Br contacts, tetra­meric Br⋯Br contacts are observed. These contacts form tetra­gonally puckered sheets parallel to (001). The CN/NC and methyl groups are grouped at the peaks and troughs. Both mol­ecules lie across crystallographic mirror planes; thus, the methyl H atoms are disordered over two sets of sites with equal occupancy. The title nitrile is a redetermination. The refinement converged at *R*[*F* ^2^ > 2σ(*F* ^2^)] = 0.020, whereas the original determination [Gleason & Britton, (1976 ▸). *Cryst. Struct. Commun.* **5**, 229–232] had *R* = 0.112.

## Abstract 2

The title crystals are isomorphous, with tetra­meric Br⋯Br contacts as the principal packing inter­action. No CN⋯Br or NC⋯Br contacts are observed.

## Chemical context   

**Figure 1:**
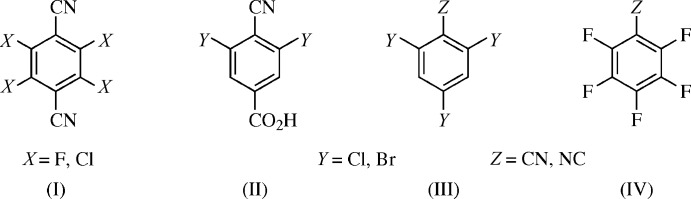
Contextual compounds.

As part of an ongoing study of cyano–halo short contacts, the para-Br atom of 2,4,6-tri­bromo­benzo­nitrile (van Rij & Britton, 1972 ▸) was replaced by a methyl group (Gleason & Britton, 1976 ▸), giving 2,6-di­bromo-4-methyl­benzo­nitrile (RCN). The methyl group was bulky enough to disrupt the planar sheet structure that was observed in the tri­bromo nitrile. As of the most recent update of the Cambridge Structural Database (CSD; Version 5.37, Feb 2017; Groom *et al.*, 2016 ▸), RCN remains the only example of a 2,6-dihalobenzo­nitrile with a methyl group at the 4-position. Most of the examples with polyatomic 4-substituents are fluorinated benzo­nitriles, with applications including tuning the fluoride affinity of phospho­ranes (Solyntjes *et al.*, 2016 ▸), study of magnetostructural correlation (Thomson *et al.*, 2012 ▸), and use as metal ligands (Díaz-Álvarez *et al.*, 2006 ▸). The chlorinated and brominated entries are either *bis*(carbo­nitriles) [(I), Fig. 1 ▸; Britton, 1981 ▸; Hirshfeld, 1984 ▸; van Rij & Britton, 1981 ▸] or 4-carb­oxy analogs [(II); Britton, 2012 ▸; Noland *et al.*, 2017 ▸]. All of these 4-substituents have stronger inter­actions than a methyl group, and exhibit different packing motifs than RCN. The comparison of corresponding nitriles and isocyanides is a rare opportunity to explore the subtle differences between mol­ecules that are both isomeric and isoelectronic. In the 2,6-dihaloaryl series, there are only three prior examples in the CSD. The tri­chloro and tri­bromo pairs [(III); Pink *et al.*, 2000 ▸; Britton *et al.*, 2016 ▸] are polytypic, and the penta­fluoro pair [(IV), Fig. 1 ▸; Bond *et al.*, 2001 ▸; Lentz & Preugschat, 1993 ▸] is isomorphous. The question arose as to whether RCN and its isocyanide (2,6-di­bromo-4-methyl­phenyl isocyanide, RNC) would be isomorphous, polytypic, or polymorphic. A single crystal of RNC and a redetermination of RCN are presented.
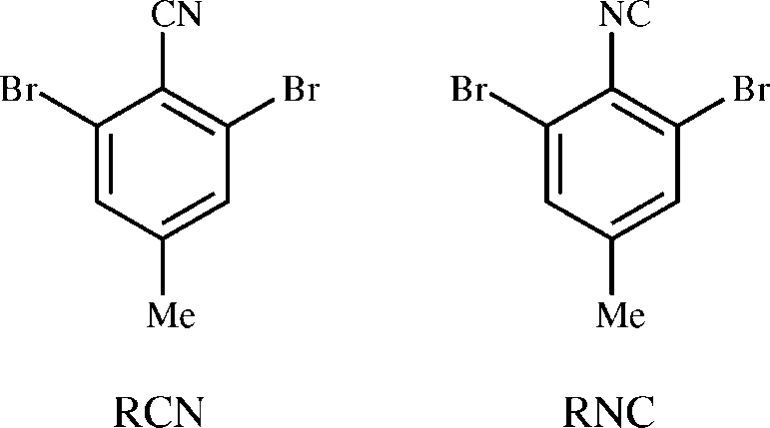


## Structural commentary   

**Figure 2:**
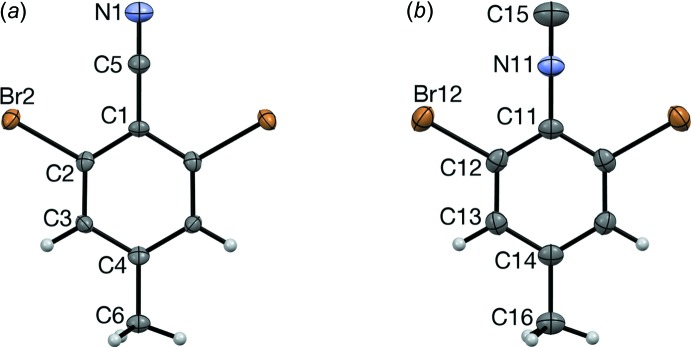
The mol­ecular structures of (*a*) RCN and (*b*) RNC, with atom labeling and displacement ellipsoids at the 50% probability level. Unlabeled atoms are generated by the (−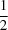 + *y*, 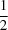 + *x*, *z*) and (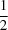 − *y*, −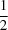 + *x*, *z*) symmetry operations, respectively. For the methyl H atoms, only one of the two mirror-related disorder sites is shown.

RNC and the redetermination of RCN are isomorphous with the original RCN structure (Gleason & Britton, 1976 ▸). The mol­ecular structures of RCN (Fig. 2 ▸ *a*) and RNC (Fig. 2 ▸ *b*) are nearly planar. The two crystals described herein were pseudo-enanti­omorphic, roughly being enanti­omorphs with swapped cyano C and N atoms, hence the reflected ellipsoid orientations between RCN and RNC. For RCN, the mean deviation from the plane of best fit for the benzene ring (C1–C4) is 0.002 (3) Å. For RNC, this deviation (C11–C14) is 0.001 (2) Å. These planes are roughly parallel to (33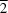).

## Supra­molecular features   

**Figure 3:**
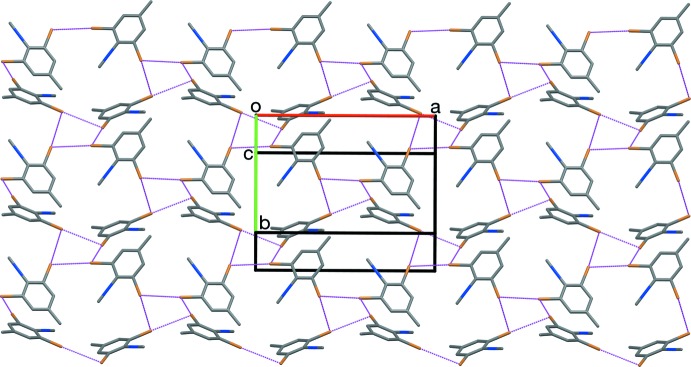
The sheet structure of RNC, viewed along [0
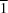
3]. The Br⋯Br contacts are represented as pink dotted lines.

The methyl group is sufficiently bulky to prevent planar ribbons or inversion dimers of the types found in the tri­bromo analogs. Instead, neighboring mol­ecules of RCN and RNC adopt a mutually inclined arrangement. The inclination between best-fit planes for adjacent mol­ecules of RCN is 38.3 (3)°, and 41.0 (2)° for RNC. This mol­ecular arrangement prevents CN⋯Br and NC⋯Br contacts, but is probably affected by the formation of 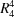
(4) rings of Br⋯Br contacts (Table 1 ▸). Each Br atom participates both as a donor (narrow C—Br⋯Br angle) and an acceptor (wide C—Br⋯Br angle). Each mol­ecule participates in two such 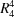
(4) rings, forming 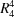
(24) rings. The result is a tetra­gonally puckered sheet structure parallel to (001) (Fig. 3 ▸). This is similar to the sheet structure reported for 2,6-di­bromo­benzo­nitrile (Britton *et al.*, 2000 ▸), although without the methyl group, the sheets were nearly planar. As future work, we plan to find whether this packing motif changes when the Br atoms are replaced with I atoms.

## Synthesis and crystallization   

**Figure 4:**
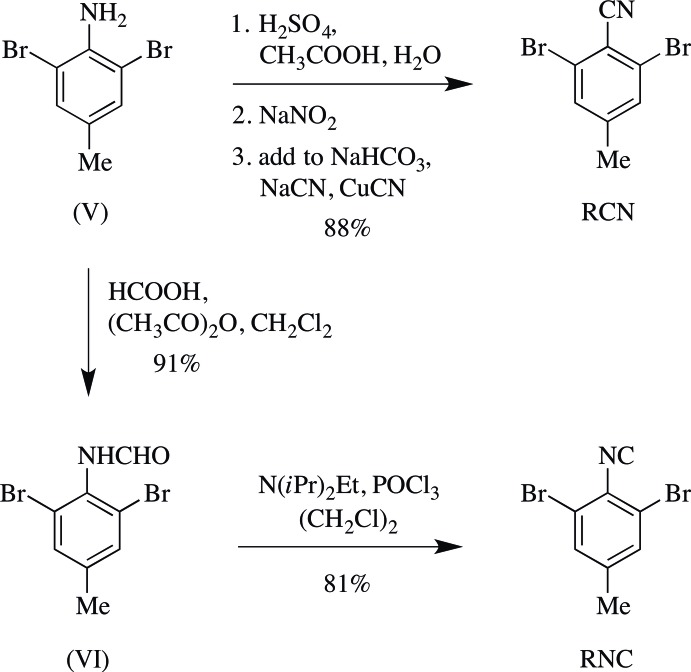
The synthesis of RCN and RNC.

The synthesis of RCN and RNC is shown in Fig. 4 ▸.

**2,6-Di­bromo-4-methyl­aniline (V)** was prepared from 4-methyl­aniline based on the work of Olivier (1926 ▸).

**RCN** was prepared from (V) (980 mg) *via* the Sandmeyer cyanation procedure described by Britton *et al.* (2016 ▸; Fig. 4 ▸), as a tan powder (898 mg, 88%). M.p. 434–435 K (lit. 429–431 K; Gleason & Britton, 1976 ▸); *R*_f_ = 0.49 (SiO_2_ in 2:1 hexa­ne–ethyl acetate); ^1^H NMR (500 MHz, CD_2_Cl_2_) *δ* 7.490 (*s*, 2H, H3*A*), 2.380 (*s*, 3H, H6*A*–*C*); ^13^C NMR (126 MHz, CD_2_Cl_2_) *δ* 147.1 (C4), 133.2 (C3), 126.6 (C2), 116.6 (C1 or C5), 116.1 (C5 or C1), 21.7 (C6); IR (KBr, cm^−1^) 3062, 2231, 1582, 1451, 1197, 857, 747; MS (EI, *m/z*) [*M*]^+^ calculated for C_8_H_5_Br_2_N 274.8763, found 274.8766.

**2,6-Di­bromo-4-methyl­formanilide (VI)** was prepared from (V) (997 mg) *via* the formyl­ation procedure described by Britton *et al.* (2016 ▸), performed at 60% scale, with di­chloro­methane instead of tetra­hydro­furan. The filter cake was recrystallized from toluene, giving white needles (1.00 g, 91%). M.p. 505–506 K; *R*_f_ = 0.27 (SiO_2_ in 2:1 hexa­ne–ethyl acetate); ^1^H NMR (500 MHz, (CD_3_)_2_SO; 2 conformers obs.) *δ* 9.993 (*s*, 1H; major), 9.743 (*d*, *J* = 10.9 Hz, 1H; minor), 8.270 (*s*, 1H; major), 8.021 (*d*, *J* = 11.1 Hz, 1H; minor), 7.623 (*s*, 2H; minor), 7.571 (*s*, 2H; major), 2.303 (*s*, 3H; both); ^13^C NMR (126 MHz, (CD_3_)_2_SO; 2 conformers obs.) *δ* 164.5 (1C; minor), 159.6 (1C; major), 140.9 (1C; minor), 140.7 (1C; major), 133.0 (2C; minor), 132.6 (2C; major), 131.9 (1C; minor), 131.8 (1C; major), 123.3 (2C; minor), 123.2 (2C; major), 19.8 (1C; both); IR (KBr, cm^−1^) 3247, 2927, 1656, 1511, 1152, 1060, 840, 747, 684; MS (ESI, *m/z*) [*M*–H]^−^ calculated for C_8_H_7_Br_2_NO 289.8822, found 289.8814.

**RNC** was prepared from (VI) (254 mg) *via* the amide dehydration procedure described by Britton *et al.* (2016 ▸), performed at 15% scale, as a beige powder (190 mg, 81%). M.p. 401–402 K; *R*_f_ = 0.53 (SiO_2_ in 3:1 hexa­ne–ethyl acetate); ^1^H NMR (400 MHz, CD_2_Cl_2_) *δ* 7.456 (*s*, 2H, H13), 2.346 (*s*, 3H, H16*A*–*C*); ^13^C NMR (101 MHz, CD_2_Cl_2_) *δ* 172.7 (C15), 142.9 (C14), 133.2 (C13), 126.0 (C11), 120.8 (C12), 21.2 (C16); IR (KBr, cm^−1^) 3061, 2922, 2850, 2118, 1654, 1586, 1451, 1384, 1064, 857, 748, 701; MS (EI, *m/z*) [*M*]^+^ calculated for C_8_H_5_Br_2_N 274.8783, found 274.8784.

**Crystallization:** RCN and RNC crystals were grown by slow evaporation of di­chloro­methane solutions under ambient conditions. Crystals were collected by suction filtration when a small portion of the original solvent remained, and then they were washed with pentane.

## Refinement   

Crystal data, data collection and structure refinement details are summarized in Table 2 ▸. A direct-methods solution was calculated, followed by full-matrix least squares/difference-Fourier cycles. All H atoms were placed in calculated positions (C—H = 0.95 or 0.98 Å) and refined as riding atoms with *U*_iso_(H) set to 1.2*U*_eq_(C) for aryl H atoms and 1.5*U*_eq_(C) for methyl H atoms. Because the mol­ecules lie across mirror planes, the methyl H atoms are disordered across two sets of sites with 1:1 occupancy.

## Supplementary Material

Crystal structure: contains datablock(s) RCN, RNC. DOI: 10.1107/S2056989017016395/lh5859sup1.cif

Structure factors: contains datablock(s) RCN. DOI: 10.1107/S2056989017016395/lh5859RCNsup2.hkl

Structure factors: contains datablock(s) RNC. DOI: 10.1107/S2056989017016395/lh5859RNCsup3.hkl

Supporting information file. DOI: 10.1107/S2056989017016395/lh5859RCNsup4.cml

Supporting information file. DOI: 10.1107/S2056989017016395/lh5859RNCsup5.cml

CCDC references: 1525809, 1525810

Additional supporting information:  crystallographic information; 3D view; checkCIF report

## Figures and Tables

**Table 1:** Contact geometry (Å, °)

C—Br⋯Br	C—Br	Br⋯Br	C—Br⋯Br
C2—Br2⋯Br2^i^	1.899 (5)	3.5575 (7)	96.8 (2)
C2—Br2⋯Br2^ii^	1.899 (5)	3.5575 (7)	176.41 (7)
C12—Br12⋯Br12^i^	1.895 (4)	3.575 (1)	97.8 (1)
C12—Br12⋯Br12^ii^	1.895 (4)	3.575 (1)	175.7 (1)

Symmetry codes: (i) 1 − *y*, *x*, 1 − *z*; (ii) *y*, 1 − *x*, 1 − *z*.

**Table 2:** Experimental details

	RCN	RNC
Crystal data		
Chemical formula	C_8_H_5_Br_2_N	C_8_H_5_Br_2_N
*M*_r_	274.95	274.95
Crystal system, space group	Tetragonal, *P* 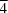 2_1_ *m*	Tetragonal, *P* 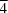 2_1_ *m*
Temperature (K)	123	173
*a*, *c* (Å)	14.6731 (5), 3.9727 (1)	14.690 (5), 4.0703 (15)
*V* (Å^3^)	855.32 (6)	878.3 (7)
*Z*	4	4
Radiation type	Cu *K*α	Mo *K*α
μ (mm^−1^)	11.46	9.16
Crystal size (mm)	0.50 × 0.07 × 0.04	0.40 × 0.14 × 0.08

Data collection		
Diffractometer	Bruker Venture PHOTON-II	Bruker APEXII CCD
Absorption correction	Multi-scan (*SADABS*; Sheldrick, 1996 ▸)	Multi-scan (*SADABS*; Sheldrick, 1996 ▸)
*T*_min_, *T*_max_	0.314, 0.754	0.255, 0.746
No. of measured, independent and observed [*I* > 2σ(*I*)] reflections	8444, 904, 902	10248, 1074, 1001
*R*_int_	0.039	0.045
(sin θ/λ)_max_ (Å^−1^)	0.624	0.652

Refinement		
*R*[*F* ^2^ > 2σ(*F* ^2^)], *wR*(*F* ^2^), *S*	0.020, 0.057, 1.27	0.023, 0.051, 1.14
No. of reflections	904	1074
No. of parameters	60	59
H-atom treatment	H-atom parameters constrained	H-atom parameters constrained
Δρ_max_, Δρ_min_ (e Å^−3^)	0.37, −0.32	0.30, −0.51
Absolute structure	Flack *x* determined using 348 quotients [(*I* ^+^)−(*I* ^−^)]/[(*I* ^+^)+(*I* ^−^)] (Parsons *et al.*, 2013 ▸)	Flack *x* determined using 381 quotients [(*I* ^+^)−(*I* ^−^)]/[(*I* ^+^)+(*I* ^−^)] (Parsons *et al.*, 2013 ▸)
Absolute structure parameter	−0.02 (3)	−0.024 (13)

Computer programs: *APEX2* and *SAINT* (Bruker, 2012 ▸), *SHELXT2014* (Sheldrick, 2015*a* ▸), *SHELXL2014* (Sheldrick, 2015*b* ▸), *Mercury* (Macrae *et al.*, 2008 ▸) and *publCIF* (Westrip, 2010 ▸).

## supplementary crystallographic information

### 3,5-Dibromo-4-methylbenzonitrile (RCN) Crystal data

**Table:**

C_8_H_5_Br_2_N	Melting point: 434 K
*M**_r_* = 274.95	Cu *K*α radiation, λ = 1.54178 Å
Tetragonal, *P*42_1_*m*	Cell parameters from 2980 reflections
*a* = 14.6731 (5) Å	θ = 4.3–74.0°
*c* = 3.9727 (1) Å	µ = 11.46 mm^−^^1^
*V* = 855.32 (6) Å^3^	*T* = 123 K
*Z* = 4	Needle, colourless
*F*(000) = 520	0.50 × 0.07 × 0.04 mm
*D*_x_ = 2.135 Mg m^−^^3^	

### 3,5-Dibromo-4-methylbenzonitrile (RCN) Data collection

**Table:**

Bruker Venture PHOTON-II diffractometer	902 reflections with *I* > 2σ(*I*)
Radiation source: ImuS micro-focus	*R*_int_ = 0.039
φ and ω scans	θ_max_ = 74.2°, θ_min_ = 6.0°
Absorption correction: multi-scan (SADABS; Sheldrick, 1996)	*h* = −18→18
*T*_min_ = 0.314, *T*_max_ = 0.754	*k* = −17→17
8444 measured reflections	*l* = −4→4
904 independent reflections	

### 3,5-Dibromo-4-methylbenzonitrile (RCN) Refinement

**Table:**

Refinement on *F*^2^	H-atom parameters constrained
Least-squares matrix: full	*w* = 1/[σ^2^(*F*_o_^2^) + 2.1952*P*] where *P* = (*F*_o_^2^ + 2*F*_c_^2^)/3
*R*[*F*^2^ > 2σ(*F*^2^)] = 0.020	(Δ/σ)_max_ = 0.001
*wR*(*F*^2^) = 0.057	Δρ_max_ = 0.37 e Å^−^^3^
*S* = 1.27	Δρ_min_ = −0.32 e Å^−^^3^
904 reflections	Extinction correction: SHELXL2014 (Sheldrick, 2015b), Fc^*^=kFc[1+0.001xFc^2^λ^3^/sin(2θ)]^-1/4^
60 parameters	Extinction coefficient: 0.0055 (4)
0 restraints	Absolute structure: Flack *x* determined using 348 quotients [(*I*^+^)-(*I*^-^)]/[(*I*^+^)+(*I*^-^)] (Parsons *et al.*, 2013)
Hydrogen site location: inferred from neighbouring sites	Absolute structure parameter: −0.02 (3)

### 3,5-Dibromo-4-methylbenzonitrile (RCN) Special details

**Table:**

Geometry. All esds (except the esd in the dihedral angle between two l.s. planes) are estimated using the full covariance matrix. The cell esds are taken into account individually in the estimation of esds in distances, angles and torsion angles; correlations between esds in cell parameters are only used when they are defined by crystal symmetry. An approximate (isotropic) treatment of cell esds is used for estimating esds involving l.s. planes.

### 3,5-Dibromo-4-methylbenzonitrile (RCN) Fractional atomic coordinates and isotropic or equivalent isotropic displacement parameters (Å^2^)

**Table:**

	*x*	*y*	*z*	*U*_iso_*/*U*_eq_	Occ. (<1)
C1	0.2722 (3)	0.7722 (3)	0.357 (2)	0.0156 (13)	
C2	0.2985 (3)	0.6837 (4)	0.2695 (13)	0.0177 (10)	
Br2	0.41762 (3)	0.64354 (3)	0.38319 (19)	0.0222 (2)	
C3	0.2404 (3)	0.6240 (3)	0.1067 (15)	0.0185 (9)	
H3A	0.2605	0.5645	0.0486	0.022*	
C4	0.1517 (3)	0.6517 (3)	0.0283 (18)	0.0189 (15)	
C5	0.3328 (4)	0.8328 (4)	0.532 (2)	0.0223 (17)	
N1	0.3806 (3)	0.8806 (3)	0.676 (2)	0.0309 (17)	
C6	0.0866 (3)	0.5866 (3)	−0.138 (2)	0.0212 (13)	
H6A	0.0306	0.6190	−0.1991	0.032*	0.5
H6B	0.0720	0.5369	0.0175	0.032*	0.5
H6C	0.1148	0.5615	−0.3417	0.032*	0.5

### 3,5-Dibromo-4-methylbenzonitrile (RCN) Atomic displacement parameters (Å^2^)

**Table:**

	*U*^11^	*U*^22^	*U*^33^	*U*^12^	*U*^13^	*U*^23^
C1	0.0183 (18)	0.0183 (18)	0.010 (3)	−0.003 (2)	0.000 (2)	0.000 (2)
C2	0.015 (2)	0.019 (2)	0.019 (3)	0.0001 (19)	0.0034 (19)	0.0025 (19)
Br2	0.0147 (3)	0.0236 (3)	0.0282 (3)	0.00002 (17)	−0.0017 (2)	0.0042 (3)
C3	0.018 (2)	0.015 (2)	0.022 (2)	−0.0001 (17)	0.002 (3)	−0.003 (2)
C4	0.018 (2)	0.018 (2)	0.020 (4)	−0.005 (3)	0.0031 (18)	0.0031 (18)
C5	0.022 (2)	0.022 (2)	0.024 (4)	−0.002 (3)	0.000 (2)	0.000 (2)
N1	0.031 (2)	0.031 (2)	0.030 (4)	−0.009 (3)	−0.003 (2)	−0.003 (2)
C6	0.023 (2)	0.023 (2)	0.017 (3)	−0.005 (3)	0.000 (2)	0.000 (2)

### 3,5-Dibromo-4-methylbenzonitrile (RCN) Geometric parameters (Å, º)

**Table:**

C1—C2^i^	1.399 (6)	C4—C3^i^	1.398 (6)
C1—C2	1.399 (6)	C4—C6	1.505 (10)
C1—C5	1.437 (10)	C5—N1	1.145 (11)
C2—C3	1.383 (8)	C6—H6A	0.9800
C2—Br2	1.899 (5)	C6—H6B	0.9800
C3—C4	1.398 (6)	C6—H6C	0.9800
C3—H3A	0.9500		
			
C2^i^—C1—C2	116.8 (7)	C3^i^—C4—C6	120.3 (3)
C2^i^—C1—C5	121.6 (3)	C3—C4—C6	120.3 (3)
C2—C1—C5	121.6 (3)	N1—C5—C1	179.0 (9)
C3—C2—C1	122.3 (5)	C4—C6—H6A	109.5
C3—C2—Br2	118.8 (4)	C4—C6—H6B	109.5
C1—C2—Br2	118.9 (4)	H6A—C6—H6B	109.5
C2—C3—C4	119.6 (5)	C4—C6—H6C	109.5
C2—C3—H3A	120.2	H6A—C6—H6C	109.5
C4—C3—H3A	120.2	H6B—C6—H6C	109.5
C3^i^—C4—C3	119.5 (6)		
			
C2^i^—C1—C2—C3	−0.5 (10)	C1—C2—C3—C4	−0.7 (9)
C5—C1—C2—C3	178.7 (7)	Br2—C2—C3—C4	178.5 (5)
C2^i^—C1—C2—Br2	−179.7 (4)	C2—C3—C4—C3^i^	1.9 (11)
C5—C1—C2—Br2	−0.5 (9)	C2—C3—C4—C6	−178.0 (6)

Symmetry code: (i) *y*−1/2, *x*+1/2, *z*.

### 2,6-Dibromo-4-methylphenyl isocyanide (RNC) Crystal data

**Table:**

C_8_H_5_Br_2_N	Melting point: 401 K
*M**_r_* = 274.95	Mo *K*α radiation, λ = 0.71073 Å
Tetragonal, *P*42_1_*m*	Cell parameters from 2995 reflections
*a* = 14.690 (5) Å	θ = 2.8–26.9°
*c* = 4.0703 (15) Å	µ = 9.16 mm^−^^1^
*V* = 878.3 (7) Å^3^	*T* = 173 K
*Z* = 4	Needle, colourless
*F*(000) = 520	0.40 × 0.14 × 0.08 mm
*D*_x_ = 2.079 Mg m^−^^3^	

### 2,6-Dibromo-4-methylphenyl isocyanide (RNC) Data collection

**Table:**

Bruker APEXII CCD diffractometer	1001 reflections with *I* > 2σ(*I*)
Radiation source: sealed tube	*R*_int_ = 0.045
φ and ω scans	θ_max_ = 27.6°, θ_min_ = 2.0°
Absorption correction: multi-scan (SADABS; Sheldrick, 1996)	*h* = −18→19
*T*_min_ = 0.255, *T*_max_ = 0.746	*k* = −19→19
10248 measured reflections	*l* = −5→5
1074 independent reflections	

### 2,6-Dibromo-4-methylphenyl isocyanide (RNC) Refinement

**Table:**

Refinement on *F*^2^	Hydrogen site location: inferred from neighbouring sites
Least-squares matrix: full	H-atom parameters constrained
*R*[*F*^2^ > 2σ(*F*^2^)] = 0.023	*w* = 1/[σ^2^(*F*_o_^2^) + (0.0267*P*)^2^ + 0.0073*P*] where *P* = (*F*_o_^2^ + 2*F*_c_^2^)/3
*wR*(*F*^2^) = 0.051	(Δ/σ)_max_ < 0.001
*S* = 1.14	Δρ_max_ = 0.30 e Å^−^^3^
1074 reflections	Δρ_min_ = −0.51 e Å^−^^3^
59 parameters	Absolute structure: Flack *x* determined using 381 quotients [(*I*^+^)-(*I*^-^)]/[(*I*^+^)+(*I*^-^)] (Parsons *et al.*, 2013)
0 restraints	Absolute structure parameter: −0.024 (13)

### 2,6-Dibromo-4-methylphenyl isocyanide (RNC) Special details

**Table:**

Geometry. All esds (except the esd in the dihedral angle between two l.s. planes) are estimated using the full covariance matrix. The cell esds are taken into account individually in the estimation of esds in distances, angles and torsion angles; correlations between esds in cell parameters are only used when they are defined by crystal symmetry. An approximate (isotropic) treatment of cell esds is used for estimating esds involving l.s. planes.

### 2,6-Dibromo-4-methylphenyl isocyanide (RNC) Fractional atomic coordinates and isotropic or equivalent isotropic displacement parameters (Å^2^)

**Table:**

	*x*	*y*	*z*	*U*_iso_*/*U*_eq_	Occ. (<1)
Br12	0.64344 (3)	0.58324 (2)	0.38265 (14)	0.03268 (14)	
N11	0.8281 (2)	0.6719 (2)	0.5307 (11)	0.0278 (11)	
C11	0.7708 (2)	0.7292 (2)	0.3538 (15)	0.0228 (11)	
C12	0.6845 (3)	0.7013 (3)	0.2671 (9)	0.0245 (8)	
C13	0.6256 (2)	0.7587 (2)	0.0981 (10)	0.0243 (8)	
H13A	0.5664	0.7382	0.0402	0.029*	
C14	0.6535 (3)	0.8465 (3)	0.0132 (13)	0.0237 (11)	
C15	0.8753 (3)	0.6247 (3)	0.6829 (18)	0.0474 (18)	
C16	0.5894 (3)	0.9106 (3)	−0.1624 (14)	0.0328 (12)	
H16A	0.5318	0.8793	−0.2065	0.049*	0.5
H16B	0.6169	0.9300	−0.3703	0.049*	0.5
H16C	0.5781	0.9640	−0.0241	0.049*	0.5

### 2,6-Dibromo-4-methylphenyl isocyanide (RNC) Atomic displacement parameters (Å^2^)

**Table:**

	*U*^11^	*U*^22^	*U*^33^	*U*^12^	*U*^13^	*U*^23^
Br12	0.0337 (2)	0.02125 (19)	0.0431 (2)	−0.00062 (15)	0.0071 (2)	0.0013 (2)
N11	0.0268 (15)	0.0268 (15)	0.030 (3)	0.009 (2)	0.0005 (13)	−0.0005 (13)
C11	0.0238 (15)	0.0238 (15)	0.021 (3)	0.0067 (19)	0.0031 (16)	−0.0031 (16)
C12	0.0267 (19)	0.0202 (18)	0.027 (2)	0.0008 (16)	0.0080 (16)	−0.0028 (15)
C13	0.0214 (17)	0.0240 (17)	0.0273 (19)	0.0001 (14)	0.0017 (18)	−0.0043 (19)
C14	0.0245 (16)	0.0245 (16)	0.022 (3)	0.007 (2)	0.0038 (14)	−0.0038 (14)
C15	0.048 (2)	0.048 (2)	0.046 (5)	0.016 (3)	−0.002 (2)	0.002 (2)
C16	0.0329 (18)	0.0329 (18)	0.033 (3)	0.009 (3)	−0.0013 (18)	0.0013 (18)

### 2,6-Dibromo-4-methylphenyl isocyanide (RNC) Geometric parameters (Å, º)

**Table:**

Br12—C12	1.896 (4)	C13—H13A	0.9500
N11—C15	1.161 (8)	C14—C13^i^	1.396 (4)
N11—C11	1.391 (7)	C14—C16	1.511 (7)
C11—C12	1.379 (5)	C16—H16A	0.9800
C11—C12^i^	1.379 (5)	C16—H16B	0.9800
C12—C13	1.389 (6)	C16—H16C	0.9800
C13—C14	1.396 (4)		
			
C15—N11—C11	178.9 (6)	C13—C14—C13^i^	118.7 (5)
C12—C11—C12^i^	118.7 (5)	C13—C14—C16	120.6 (2)
C12—C11—N11	120.6 (3)	C13^i^—C14—C16	120.6 (2)
C12^i^—C11—N11	120.6 (3)	C14—C16—H16A	109.5
C11—C12—C13	121.3 (4)	C14—C16—H16B	109.5
C11—C12—Br12	120.0 (3)	H16A—C16—H16B	109.5
C13—C12—Br12	118.7 (3)	C14—C16—H16C	109.5
C12—C13—C14	120.0 (4)	H16A—C16—H16C	109.5
C12—C13—H13A	120.0	H16B—C16—H16C	109.5
C14—C13—H13A	120.0		
			
C12^i^—C11—C12—C13	−0.5 (8)	C11—C12—C13—C14	−0.1 (6)
N11—C11—C12—C13	178.2 (4)	Br12—C12—C13—C14	179.2 (3)
C12^i^—C11—C12—Br12	−179.8 (3)	C12—C13—C14—C13^i^	0.7 (7)
N11—C11—C12—Br12	−1.1 (7)	C12—C13—C14—C16	−178.3 (4)

Symmetry code: (i) −*y*+3/2, −*x*+3/2, *z*.
